# Decoupled Drivers of Phylogenetic Diversity and Community Assembly Signals Across Forest Types in a Temperate Forest, South Korea

**DOI:** 10.3390/life16020301

**Published:** 2026-02-10

**Authors:** Chang-Bae Lee

**Affiliations:** 1 Department of Forest Resources, Kookmin University, 77 Jeongneungro, Seongbukgu, Seoul 02707, Republic of Korea; kecolee@kookmin.ac.kr; 2 Department of Climate Technology Convergence, Kookmin University, 77 Jeongneungro, Seongbukgu, Seoul 02707, Republic of Korea; 3 Forest Carbon Graduate School, Kookmin University, 77 Jeongneungro, Seongbukgu, Seoul 02707, Republic of Korea

**Keywords:** community-weighted mean, phylogenetic diversity, phylogenetic community structure, species richness, specific leaf area

## Abstract

Phylogenetic metrics can separate two complementary biodiversity dimensions: the amount of evolutionary history retained in a community (Faith’s phylogenetic diversity, PD) and community assembly signals expressed as departures from null expectations in phylogenetic relatedness (standardized effect size of mean pairwise phylogenetic distance, SES.MPD). However, at the individual-forest scale—where conservation and management decisions are implemented—the key drivers and linked pathways controlling these two dimensions often remain unclear. Here, PD and SES.MPD were quantified for 96 20 × 20 m forest plots spanning broadleaved, conifer, and subalpine forests on Mt. Gariwang, South Korea. Community phylogenies were generated and related to elevation, stand age class, soil fertility, species richness, and community-weighted mean (CWM) traits (specific leaf area, SLA; wood density, WD) using information-theoretic multimodel inference and piecewise structural equation modeling. PD and SES.MPD differed significantly among forest types, but were governed by distinct controls. PD was most strongly and negatively associated with CWM.WD, indicating that dominance by high–wood-density strategies coincided with reduced retained evolutionary history. In contrast, SES.MPD was primarily negatively associated with CWM.SLA and species richness, with soil fertility influencing SES.MPD indirectly via SLA; stand age class showed limited explanatory power. Overall, these results demonstrate decoupled drivers of evolutionary-history retention versus assembly-related coexistence structure and identify management-relevant levers at the individual-forest scale, highlighting the importance of trait dominance and soil–trait pathways in addition to forest type.

## 1. Introduction

Global environmental changes—such as climate change and biodiversity loss—affect ecosystem functioning and biodiversity simultaneously across both small and large spatial scales [1,2]. Under these natural and anthropogenic disturbances, understanding the spatiotemporal processes and mechanisms underlying changes in community composition and structure is essential for conserving and managing the species that constitute ecological communities [3]. Consequently, research on the processes and mechanisms governing community assembly has long been a central topic in ecology [3,4]. More recently, this field has rapidly expanded beyond purely taxon-based approaches toward the use of phylogenetic diversity and phylogenetic community structure, which incorporates evolutionary history and relatedness among species to interpret community assembly mechanisms and link them to ecosystem functions and services [5,6,7]. Because phylogenetic relationships reflect shared evolutionary history and may also capture similarities in ecologically relevant traits when those traits are phylogenetically conserved, the phylogenetic approaches provide a useful framework for indirectly inferring the ecological processes shaping community patterns, including environmental filtering, interspecific interactions, and dispersal limitation [5,6,8]. However, inferring processes from phylogenetic patterns is not always straightforward because similar phylogenetic signatures can arise from different mechanisms (e.g., filtering vs. competitive sorting), and their interpretation can depend on the strength of phylogenetic signal in key traits and on the choice of null models and spatial scale [5,8,9,10].

According to community assembly theory, realized community structure emerges from the interplay between deterministic processes (e.g., environmental filtering, competition, and niche partitioning) and stochastic processes (e.g., dispersal limitation, stochastic colonization and local extinction, and disturbance), with their relative importance varying along environmental gradients and across spatial scales [3,4,9,10]. This perspective has also been summarized as a conceptual synthesis that explains diverse community patterns through a small set of fundamental processes [3].

Phylogenetic diversity and community structure can be quantified using several metrics, among which Faith’s phylogenetic diversity (PD) and the standardized effect size of mean pairwise phylogenetic distance (SES.MPD) are among the most widely used [5,11,12,13]. Faith’s PD represents the total phylogenetic branch length (i.e., the amount of evolutionary history) encompassed by a community, whereas SES.MPD is useful for determining whether the observed mean phylogenetic distance is more clustered or more overdispersed than expected under a null model [5,12]. Thus, PD reflects the absolute amount of evolutionary history contained in a community, whereas SES.MPD more directly captures the relative deviation in phylogenetic relatedness (i.e., the community assembly signal) given the local species richness and species pool; the two metrics therefore provide complementary information [5,13].

In forest ecosystems, phylogenetic structure can be tightly linked to environmental gradients (e.g., elevation and topography), edaphic conditions (e.g., soil moisture and nutrient status), and stand development and disturbance legacies represented by stand age, as well as to dominant community strategies (e.g., vertical competition and growth strategies reflected by maximum height) [6,8]. For example, chronosequence studies have reported that phylogenetic clustering tends to weaken as stand age increases and environmental quality improves, suggesting a transition from strong early filtering toward a reorganization of coexistence structure over time [14]. Along elevational gradients, phylogenetic structure can also shift markedly, and soil variables such as soil total nitrogen have been identified as key predictors of phylogenetic variation [15]. In the last ten years, a growing body of research has refined these interpretations: studies have documented elevational shifts in the phylogenetic structure of woody plant communities (e.g., increasing tendencies toward overdispersion with elevation), highlighted soil nitrogen as a core explanatory variable, and proposed coupled pathways linking phylogenetic diversity, trait-based community metrics (e.g., community-weighted means, CWM), and environmental drivers—thereby connecting phylogenetic structure to ecosystem functioning [15,16,17]. Other studies have jointly evaluated PD and phylogenetic community structure across elevational bands to test competing macroecological hypotheses, underscoring both the generality and context dependence of elevation–environment–phylogenetic relationships [15,18]. Nevertheless, two conceptual gaps remain prominent for temperate mountain forests: (i) whether trait dominance (e.g., acquisitive vs. conservative strategies captured by CWM traits) primarily constrains retained evolutionary history (PD) or instead shapes assembly signals (SES.MPD), and (ii) whether edaphic conditions act mainly as direct drivers or indirectly through trait-mediated pathways.

Despite these advances, many previous studies have been strongest in deriving average patterns from broad-scale datasets at regional or national extents, whereas forest conservation, management, and restoration decisions are typically made at the scale of individual forests (e.g., a specific mountain, watershed, or management unit). Even within the same climatic zone and forest type, individual forests or stands may differ in microtopography and soil heterogeneity, disturbance and management histories, and constraints on the local species pool and dispersal processes; consequently, the drivers and pathways (direct and indirect effects) shaping phylogenetic structure can differ substantially [2,10]. Therefore, to translate broad-scale generalities into actionable guidance for on-the-ground management, it is essential to diagnose phylogenetic structure at the individual-forest scale and to identify how controlling factors operate through linked pathways. In this sense, broad-scale comparative studies (e.g., evaluating how abiotic drivers and species’ niche characteristics regulate diversity metrics across forest types) and mechanism-focused analyses within individual forests are complementary. In particular, an individual-forest framework can reveal whether management-relevant levers differ for conserving evolutionary history (PD) versus maintaining coexistence structure and assembly signals (SES.MPD), which are often implicitly assumed to respond similarly.

Moreover, the key variables commonly considered in studies of community composition and structure—such as elevation, stand age, soil nutrients, species diversity, and functional traits—are not independent but are often connected through cascading and mediating relationships. Accordingly, simple regressions or correlation analyses alone have limited ability to disentangle what directly alters phylogenetic structure from what operates indirectly through other factors. This motivates the need for analytical approaches that can quantify such complex pathways and estimate direct and indirect effects simultaneously, thereby improving the transparency of causal interpretation [19,20]. This need is especially acute in temperate mountain forests, where elevation covaries with climate and soils, and where soil–trait linkages can generate indirect effects that may be mistaken for direct environmental filtering if pathways are not explicitly evaluated.

Against this background, the present study was conducted in a representative temperate forest in South Korea, aiming to: (1) quantify phylogenetic diversity and phylogenetic community structure using PD and SES.MPD; (2) evaluate their relationships with elevation, stand age, soil nutrients, species richness, and the dominance of key trait metrics within communities (i.e., specific leaf area and maximum height); and (3) use a linked-pathway framework to simultaneously identify the direct and indirect effects and causal relationships among these variables. Specifically, the analysis tests whether (i) PD and SES.MPD are controlled by the same versus decoupled drivers, (ii) trait dominance provides stronger explanatory power than stand age for phylogenetic patterns within a single temperate mountain forest, and (iii) soil fertility influences phylogenetic metrics primarily through trait-mediated pathways rather than direct effects. Through this approach, this study seeks to provide phylogenetically informed evidence for prioritizing which factors should be monitored and targeted for intervention in forest conservation, management, and restoration at the individual-forest scale.

## 2. Materials and Methods

### 2.1. Study Area and Field Survey

**Figure 1:**
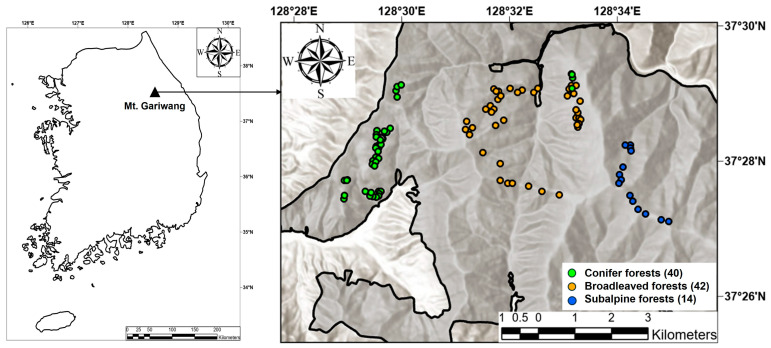
Location and distribution of 96 study plots (400 m^2^) in Mt. Gariwang, South Korea.

This study was conducted on Mt. Gariwang, located across Pyeongchang-gun and Jeongseon-gun, Gangwon Province, South Korea (Figure 1). Mt. Gariwang is widely recognized as a representative montane ecosystem in Korea, where temperate deciduous broadleaved forests are prevalent. The mountain reaches 1561 m a.s.l., with mean annual temperature ranging from 7.1 to 13.5 °C and mean annual precipitation from 1308 to 1659 mm [21]. Forests dominated by *Quercus mongolica* are common, with conifer and mixed forests (e.g., including *Pinus densiflora*) also present. Subalpine zones include forests composed of cold-adapted species such as *Abies nephrolepis*, *Abies holophylla*, *Taxus cuspidata*, *Pinus koraiensis*, and *Betula ermanii* [21].

Vegetation surveys were carried out across three forest types during the peak biomass period (July–August) in 2020–2021: (1) broadleaved forests dominated by *Q. mongolica*, other *Quercus* spp., and other broadleaved species; (2) conifer forests including *Larix kaempferi*, *P. densiflora*, and *P. koraiensis*; and (3) subalpine forests characterized by the co-occurrence of *A. holophylla*, *B. ermanii*, and *Thuja* spp. For each dominant-species stand type, 14 plots were established, except for the *P. koraiensis* stands (12 plots). In total, 96 plots were surveyed: 42 in broadleaved forests, 40 in conifer forests, and 14 in subalpine forests. All plots were 20 m × 20 m (400 m^2^). Within each plot, all woody plants with diameter at breast height (DBH) ≥ 2 cm were identified to species, and DBH and tree height were measured and recorded.

### 2.2. Quantification of Phylogenetic Diversity and Community Structure

To analyze phylogenetic community structure in each plot, a phylogenetic tree was constructed using the V.PhyloMaker2 package in R version 4.1.2 [22] based on the tree species recorded in the plots (Figure S1). This phylogeny was used to calculate phylogenetic diversity and phylogenetic community structure in a plot.

For each plot, phylogenetic diversity was quantified as Faith’s phylogenetic diversity (hereafter PD), and phylogenetic community structure was quantified as the standardized effect size of mean pairwise phylogenetic distance (SES.MPD) [5,11,23]. PD represents the absolute amount of evolutionary history contained in a community, whereas SES.MPD captures phylogenetic relatedness among co-occurring species and is widely used to infer phylogenetic community structure [5]. PD was calculated as the sum of phylogenetic branch lengths connecting all species present in a plot [11]. SES.MPD was calculated as:

$$SES.MPD=\frac{{MPD}_{obs}-{meanMDP}_{rand}}{{SD}_{rand}}$$

where MPD_obs_ is the mean phylogenetic distance among all possible species pairs within a plot, and meanMPD_rand_ and SD_rand_ are the mean and standard deviation of MPD values computed from 1000 randomized assemblages [5,24]. Random assemblages were generated by randomly shuffling species labels across the tips of the phylogeny. This randomization method preserves the observed community matrix while randomizing phylogenetic relationships among species; thus, species richness, species occurrence frequencies, and spatial occupancy patterns are maintained in each randomization [5,12,25,26]. Negative SES.MPD values indicate phylogenetic clustering, whereas positive values indicate phylogenetic overdispersion [5,6,7]. PD and SES.MPD were computed using the picante package in R version 4.1.2.

### 2.3. Quantification of Biotic and Abiotic Factors

Biotic factors potentially influencing phylogenetic community structure were represented by species richness (SR), the community-weighted mean (CWM) of specific leaf area (SLA), the CWM of maximum tree height (MH), and the CWM of wood density (WD). Species richness provides a baseline for interpreting phylogenetic structure by indicating how much phylogenetic space is potentially occupied by the community [24]. CWM is a community-level summary that integrates interspecific variation in functional traits, allowing inference about which trait strategies are favored during community assembly [24,27]. SLA, MH, and WD were selected because SLA reflects resource-use strategy (e.g., photosynthetic capacity, growth rate, and nutrient cycling), whereas MH is a size-related trait associated with canopy structure, light competition, and long-term biomass accumulation. WD captures variation in mechanical strength and life-history strategy (growth–survival trade-offs) and is closely linked to hydraulic safety and carbon storage [24]. Trait data were obtained from a dataset compiled by the author’s laboratory; for each species, specific leaf area and wood density were measured for at least three individuals sampled during July–August [27]. Trait measurements followed the standardized protocols described in Pérez-Harguindeguy et al. [28]. CWMs were calculated as:

$${CWM}_{x}=\sum_{i=1}^{n}p_{i}t_{i}$$

where x denotes the focal trait (SLA or MH), n is the number of tree species in the plot, p_i_ is the relative basal area of species i (i.e., basal area of species i divided by the total basal area of all tree species in the plot), and t_i_ is the mean trait value of species i.

Abiotic factors included elevation, mean annual temperature (MAT) and mean annual precipitation (MAP), soil properties, and stand age class, which are commonly considered key filters associated with topography, climate, soil conditions, and forest succession. Elevation was extracted from a digital elevation model provided by the National Geographic Information Institute (NGII). MAT and MAP were extracted from a digital climate map developed by the National Institute of Forest Science [21,26]. Soil variables included total nitrogen (TN), available phosphorus (P), and cation exchange capacity (CEC). These variables capture complementary components of forest soil fertility [28,29]: TN and P represent key nutrient pools and availability that constrain plant growth, whereas CEC reflects the soil’s capacity to retain exchangeable nutrient cations and buffer nutrient supply. Accordingly, TN, P, and CEC are widely used as chemical indicators in assessments of forest soil quality, soil health, and soil fertility [28,29,30,31]. For soil analyses, samples were collected during July–August (2020–2021) from five points within each plot at 0–30 cm depth, composited into a single sample per plot, and analyzed by the Korea Forestry Promotion Institute. Stand age class, representing successional and developmental stage, was obtained from forest type maps (map.forest.go.kr accessed on 5 November 2025) provided by the Korea Forest Service [28]. Stand age class is defined in 10-year intervals and is determined either by tree-ring analysis of increment cores collected from dominant and co-dominant canopy trees (≥50% of the canopy layer) or by stand history records. For statistical analyses, stand age class was treated as an ordered numeric variable.

### 2.4. Statistical Analyses

Prior to statistical analyses, all variables were log- or square-root-transformed as needed to improve linearity and normality, and then standardized to reduce unit differences and enhance comparability among predictors (Table S1). To minimize multicollinearity, correlation analyses were conducted among explanatory variables (Figure S2), and elevation was found to be highly correlated with MAT and MAP (|r| > 0.8); therefore, MAT and MAP were excluded from the final analyses. Among the three soil variables such as TN, P, and CEC, pairwise correlations were moderate to strong (TN–CEC, r = 0.80; P–CEC, r = 0.80; TN–P, r = 0.60), indicating substantial multicollinearity if included simultaneously as separate predictors. Accordingly, principal component analysis (PCA) was applied to the three variables to summarize their shared variation into a composite soil-fertility gradient (Table S2). The first principal component (PC1), which explained 81.6% of the total variance, was used as a composite soil predictor and was termed PC1_soil. In addition, because CWM.SLA and CWM.MH were highly correlated (|r| > 0.8), CWM.MH was excluded, and CWM.SLA and CWM.WD were retained for subsequent analyses.

PCA was also used to visualize multivariate patterns among the three pre-defined forest types (i.e., broadleaved, conifer, and subalpine forests) based on the explanatory variables. To formally test whether the multivariate predictor composition differed among forest types, a distance-based PERMANOVA (9999 permutations) was performed on a Euclidean distance matrix computed from standardized explanatory variables using adonis2 function in R version 4.1.2. Homogeneity of multivariate dispersions was evaluated using betadisper function in R version 4.1.2 to ensure that PERMANOVA results were not driven by dispersion differences.

To evaluate differences in PD and SES.MPD among forest types, one-way ANOVA was performed, followed by Tukey’s HSD post hoc tests for pairwise comparisons. To support inferences about phylogenetic clustering and overdispersion, one-sample t-tests were additionally conducted within each forest type to assess whether mean SES.MPD significantly deviated from zero (null expectation).

To assess the relative importance of explanatory variables for PD and SES.MPD, an information-theoretic multimodel inference approach based on AIC was applied [32,33,34]. Candidate models were specified as linear mixed-effects models with a random effect for forest type and were generated as all possible subsets of the fixed-effect terms from an a priori global model. Forest type was treated as a random effect (grouping factor) to account for forest-type-level clustering while estimating the effects of continuous predictors [35,36]. Model sets were ranked using AIC, and models with ΔAIC ≤ 2 were retained as similarly supported candidates [34,37,38]. Model averaging was then performed across this retained set using Akaike weights to account for model-selection uncertainty and to obtain model-averaged standardized coefficients (and unconditional uncertainty) for each predictor [28,34].

Relative importance of each predictor was calculated by summing the absolute values of standardized coefficients (|β|) across the averaged models and then dividing each predictor’s summed |β| by the total summed |β| to yield proportional importance values [36,37].

To explain linked pathways among explanatory variables and phylogenetic diversity/community structure, piecewise structural equation modeling (pSEM) was conducted based on a conceptual model (Figure S3) [20]. A full model including all hypothesized paths between explanatory variables and phylogenetic responses was first specified. Non-significant paths were then removed based on d-separation tests, and the resulting model was selected as the final pSEM. To quantify the influence of forest type, pSEM was combined with mixed-effects modeling by including forest type as a random effect. Model fit was evaluated using Fisher’s C statistic, its associated *p*-value, and AIC.

Because Faith’s phylogenetic diversity (PD) is intrinsically dependent on species richness (i.e., PD increases with the number of species by construction because it sums total phylogenetic branch length), species richness was excluded as a predictor from the PD analyses in both the multimodel inference and the pSEM to avoid a trivial and inherent dependency. In contrast, species richness was retained as a predictor for SES.MPD because SES.MPD is a standardized effect size of mean pairwise phylogenetic distance under a null model and is not trivially determined by species number.

PERMANOVA, multimodel inference and pSEM analyses were conducted in R version 4.1.2 using the vegan, MuMIn and piecewiseSEM packages.

## 3. Results

**Figure 2:**
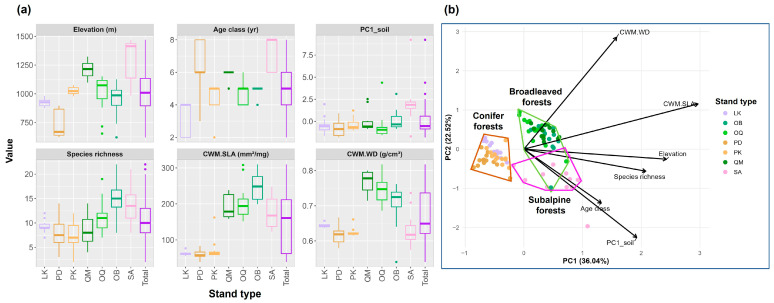
Summary statistics of (**a**) explanatory variables across stand types on Mt. Gariwang, South Korea, and (**b**) the principal component analysis (PCA) based on these variables. The PCA ordination of explanatory variables visualizes differences among the pre-defined stand groups, with patterns consistent with three broader forest categories: broadleaved forests (QM, OQ, OB), conifer forests (LK, PD, PK), and subalpine forests (SA). PCA is presented for visualization only, and group differences were formally tested using PERMANOVA (see Section 3). Abbreviations: CWM, community weighted mean; SLA, specific leaf area; WD, wood density; PC1_soil, the first principal component axis derived from a PCA of soil properties; LK, *Larix kaempferi* stands; PD, *Pinus densiflora* stands; PK, *Pinus koraiensis* stands; QM, *Quercus mongolica* stands; OQ, other *Quercus* spp. stands; OB, other broadleaved stands; SA, subalpine stands.

PCA ordination of the explanatory variables visualized multivariate differences among the pre-defined forest types (Figure 2), with patterns consistent with separation among broadleaved, conifer, and subalpine forests. PERMANOVA confirmed that forest types differed significantly in multivariate predictor composition (pseudo-F = 38.885, R^2^ = 0.455, *p* < 0.001). Multivariate dispersions did not differ among forest types (betadisper permutation test: *p* = 0.267), supporting that the PERMANOVA result reflects differences in group centroids. Pairwise PERMANOVA indicated significant differences for all forest-type pairs (Holm-adjusted *p* < 0.001; R^2^ = 0.29–0.44).

**Figure 3:**
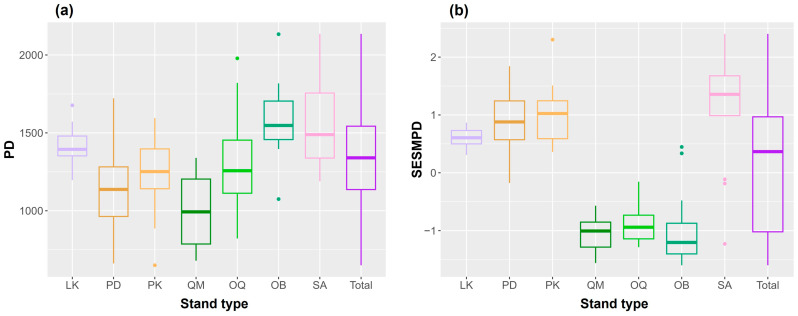
Boxplots showing differences in (**a**) phylogenetic diversity (PD) and (**b**) phylogenetic community structure (SES.MPD; standardized effect size of mean pairwise phylogenetic distance) among stand types on Mt. Gariwang, South Korea. Abbreviations are provided in Figure 2.

In comparisons of PD and SES.MPD among forest types (Figure 3), both PD (ANOVA: F = 4.32, *p* = 0.016) and SES.MPD (ANOVA: F = 126, *p* < 0.001) differed significantly among the three forest types. Tukey’s HSD post hoc test for PD showed that subalpine forests differed from both broadleaved (*p* = 0.026) and conifer forests (*p* = 0.016), whereas broadleaved and conifer forests did not differ (*p* = 0.959). For SES.MPD, subalpine forests differed from broadleaved forests (*p* < 0.001) but not from conifer forests (*p* = 0.177), while broadleaved and conifer forests differed significantly (*p* < 0.001). In addition, one-sample tests against zero indicated significant phylogenetic clustering in broadleaved forests (mean SES.MPD = −0.96, t = −13.57, *p* < 0.001) and significant phylogenetic overdispersion in conifer forests (mean SES.MPD = 0.82, t = 11.01, *p* < 0.001) and subalpine forests (mean SES.MPD = 1.14, t = 4.20, *p* = 0.001).

**Figure 4:**
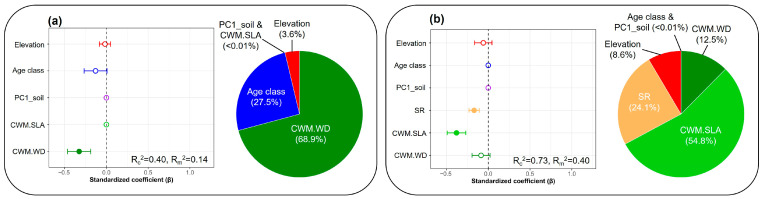
Relative contributions of abiotic and biotic drivers to (**a**) phylogenetic diversity and (**b**) phylogenetic community structure based on multimodel inference tests on Mt. Gariwang, South Korea. Abbreviations are provided in Figure 2.

Multimodel inference indicated that CWM.WD had the strongest influence on phylogenetic diversity (PD) (Figure 4a). Phylogenetic community structure (SES.MPD) was negatively associated with CWM.SLA and species richness (SR) (Figure 4b). In addition, forest type accounted for a substantial share of the explained variation in both responses: the contribution, estimated as the difference between conditional and marginal R^2^ (R_c_^2^ − R_m_^2^), was 0.26 for PD and 0.33 for SES.MPD, indicating that 26% and 33% of the explained variance, respectively, was attributable to forest-type-level clustering.

**Figure 5:**
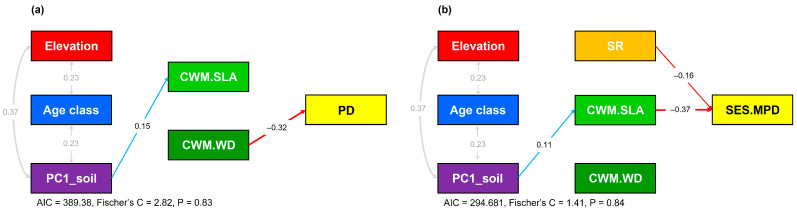
Piecewise structural equation models testing multiple pathways through which abiotic and biotic drivers influence (**a**) phylogenetic diversity and (**b**) phylogenetic community structure on Mt. Gariwang, South Korea. Solid blue and red arrows indicate significant (*p* < 0.05) positive and negative effects, respectively, and gray arrows indicate estimated covariance. Abbreviations are provided in Figure 2.

Piecewise structural equation modeling (pSEM) further clarified linked pathways among predictors (Figure 5). In the PD model, PD decreased with increasing CWM.WD (β = −0.32), while soil fertility (PC1_soil) was positively associated with CWM.SLA (β = 0.15). In the SES.MPD model, SES.MPD decreased with increasing CWM.SLA (β = −0.37) and species richness (β = −0.16) and PC1_soil influenced SES.MPD indirectly via CWM.SLA.

## 4. Discussion

This study evaluated how abiotic and biotic factors jointly regulate phylogenetic diversity (Faith’s PD) and phylogenetic community structure (SES.MPD) across forest types in Mt. Gariwang, South Korea. A key implication is that PD (the amount of evolutionary history retained) and SES.MPD (the direction and strength of phylogenetic relatedness relative to null expectations) can respond to different ecological axes and therefore should be interpreted jointly rather than interchangeably [3,4,5]. In the revised analyses, PD and SES.MPD differed significantly among forest types, yet they conveyed distinct information: PD captured forest-type differences in retained evolutionary history, whereas SES.MPD revealed contrasting assembly signals (clustering vs. overdispersion) among broadleaved versus conifer/subalpine forests.

### 4.1. Forest-Type Contrasts in PD and SES.MPD Indicate Decoupled Biodiversity Dimensions

The significant differentiation of SES.MPD among forest types—phylogenetic clustering in broadleaved forests and phylogenetic overdispersion in conifer and subalpine forests—suggests that coexistence is structured differently even within a single mountain system. Such contrasts are consistent with the general framework of phylogenetic community ecology in which clustering can emerge under strong environmental filtering when ecologically important traits are phylogenetically conserved, whereas overdispersion can arise through niche differentiation, competitive exclusion among close relatives, or the coexistence of distantly related lineages with convergent strategies [3,4,17,18,19,39]. Recent montane-gradient studies likewise emphasize that phylogenetic patterns can shift with elevation and habitat context, and that phylogenetic and functional signals may not change in parallel (e.g., functional convergence co-occurring with phylogenetic overdispersion under strong stress) [17,18,25]. Therefore, the observed clustering–overdispersion contrast among forest types is most parsimoniously interpreted as an outcome of multiple, non-exclusive mechanisms operating along topographic, microclimatic, and edaphic heterogeneity typical of temperate mountain forests [23,24].

Notably, one-sample tests against zero supported the inference that broadleaved forests were significantly clustered while conifer and subalpine forests were significantly overdispersed. This is important because conclusions about clustering/overdispersion require hypothesis testing relative to the null expectation (SES.MPD = 0), not only comparisons among groups [3,6,17]. Together, the forest-type contrasts imply that management-relevant assembly signals can vary strongly among stand types even where the overall site context is shared.

### 4.2. Wood Density as a Principal Driver of PD Suggests Trait-Mediated Filtering on Retained Evolutionary History

In multimodel inference and pSEM, PD was most strongly and negatively associated with community-weighted mean wood density (CWM.WD). Wood density is a core plant functional trait linked to growth–survival trade-offs, mechanical strength, and hydraulic safety, and it often shows substantial phylogenetic structuring across woody lineages [7,9]. A negative PD–CWM.WD relationship can be interpreted in at least two complementary ways. First, dominance by high-wood-density strategies may reflect environmental filtering (e.g., stressful microsites, resource limitation, or disturbance legacies) that favors a subset of conservative lineages, thereby reducing the breadth of evolutionary history represented locally [3,17,19]. Second, if high wood density is concentrated in particular clades within the regional species pool, increasing dominance by those clades can lower PD by narrowing the set of deep lineages co-occurring within plots, even when species numbers are not dramatically different [5,6]. Importantly, species richness was excluded from PD modeling to avoid a trivial dependency (PD increases with species number by construction), so the identified PD–CWM.WD association indicates a non-trivial, trait-linked signal beyond the mathematical richness effect [5,39].

The finding that forest type accounted for a substantial portion of PD variation (random-effect contribution in mixed modeling) further indicates that broad forest categories reflect consistent differences in the combination of strategies and lineages retained at the stand scale. Comparable trait-mediated controls on phylogenetic diversity have been increasingly reported in forest systems, where trait composition and edaphic context jointly shape which lineages can persist and dominate [14,25,26,40].

### 4.3. SLA, Soil Fertility, and Richness Regulate Assembly Signals Captured by SES.MPD

SES.MPD decreased with increasing CWM.SLA and (to a lesser extent) species richness, and soil fertility influenced SES.MPD indirectly through CWM.SLA. SLA is a canonical trait axis representing the leaf economics spectrum, with high SLA generally reflecting acquisitive strategies (rapid return on investment, fast growth) and low SLA reflecting conservative strategies [7]. A negative SES.MPD–CWM.SLA association implies that communities dominated by acquisitive strategies tend to be more phylogenetically clustered. This pattern is consistent with a scenario where resource-rich conditions favor repeated dominance of certain trait syndromes and lineages (filtering), and/or where competitive exclusion among similar strategies yields dominance by a subset of closely related taxa [3,18,19,41].

The indirect effect of soil fertility on SES.MPD via SLA provides an interpretable mechanism: fertility gradients can shift dominance toward acquisitive leaf strategies, and such shifts can restructure phylogenetic relatedness among co-occurring species. Similar soil–trait–community linkages have been emphasized in recent forest studies showing that soil nutrients (often nitrogen-related) and edaphic capacity to supply/retain nutrients can mediate community trait composition and diversity patterns [26,27,42]. In addition, recent work highlights that phylogenetic metrics and trait-based metrics can provide complementary (not redundant) insights into ecological mechanisms and ecosystem functions, reinforcing the value of reporting both dimensions in management-oriented forest assessments [6,28,43].

### 4.4. Limited Stand-Age Effects Highlight Context Dependence and the Role of Mediating Pathways

Stand age class did not emerge as a dominant determinant in the revised pathway models. This does not imply that succession is irrelevant; rather, it suggests that, at this site and resolution, successional influences may be expressed primarily through mediators (e.g., soils and trait composition) rather than direct age effects, and/or that the age-class map (10-year bins) may not capture fine-scale variation in disturbance history and structural development [25,26,44]. Chronosequence studies in forests have reported variable age-related trajectories in phylogenetic structure, including weakening clustering with stand development in some contexts but strong edaphic control in others, implying that age effects are often contingent on soil recovery rates, colonization processes, and management legacies [26,29]. Therefore, stand age should be interpreted as one component of a broader causal network rather than as a universally dominant predictor.

### 4.5. Management Implications at the Individual-Forest Scale

Because PD and SES.MPD responded to different levers, practical monitoring and intervention can be more targeted when both metrics are evaluated jointly. For conserving evolutionary history (PD), the results suggest prioritizing processes that maintain phylogenetically broad species representation and avoiding management trajectories that disproportionately favor a narrow set of high-wood-density lineages. In practice, this may include maintaining regeneration pathways that allow multiple lineages to establish and persist, and preventing structural or compositional simplification that concentrates dominance within a limited functional–phylogenetic subset [6,14,28,45].

For conserving coexistence structure (SES.MPD), trait composition—especially SLA—emerged as a proximate regulator, with soil fertility acting through trait shifts. This supports a soil–trait–assembly management logic: minimizing soil disturbance (erosion/compaction), maintaining organic matter and nutrient-retention capacity, and monitoring trait shifts in dominant strata can help sustain phylogenetic coexistence patterns and potentially buffer vulnerability to pests/pathogens and climatic shocks associated with overly clustered assemblages [18,27,28]. Finally, the overdispersion signal in conifer and subalpine forests suggests that conserving fine-scale habitat heterogeneity (microtopographic units, snow/wind exposure gradients, small edaphic mosaics) may be especially important because such heterogeneity can promote the coexistence of distantly related lineages even when functional strategies converge under stress [23,25].

### 4.6. Limitations and Future Directions

Several limitations merit attention. First, elevation is an integrative proxy for climate, but montane assembly is often sensitive to microclimate (slope aspect, cold-air drainage, ridge–valley contrasts, snow persistence) that may not be captured by coarse predictors; combining plot data with sensor-based microclimate or high-resolution downscaled products would strengthen inference [23,24,46]. Second, SES.MPD was standardized using a tip-label randomization (taxa.labels) null model that preserves the observed community matrix while randomizing phylogenetic relationships among species; this approach is widely used to test whether observed phylogenetic relatedness deviates from null expectations given the observed species occurrences. However, different null models (e.g., those constraining species occurrence frequencies or incorporating dispersal/environmental constraints) can yield different inferences, and future work could evaluate the robustness of assembly interpretations by comparing alternative null-model formulations. Third, PD and SES.MPD were derived from a large-scale vascular plant megaphylogeny; such megaphylogenies enable broad comparability but retain uncertainty in branch lengths and species-level relationships, suggesting value in developing regionally informed phylogenies using local barcode or genomic resources. Fourth, functional strategies were represented using community-weighted means of specific leaf area (SLA) and wood density (WD), which capture major axes of plant ecological strategy (resource economics and tissue investment), and WD was incorporated during the additional analyses to better represent conservative growth and stress-tolerance strategies. Nevertheless, these traits do not fully capture other key dimensions relevant to montane forests (e.g., cold tolerance, hydraulic traits, phenology, rooting depth, or regeneration traits), and expanding trait coverage would improve mechanistic attribution of phylogenetic patterns. Finally, intraspecific genetic diversity and population structure were not evaluated, although they can influence dispersal limitation, adaptive potential, and long-term resilience; integrating genetic layers into trait–environment–phylogeny frameworks represent a promising next step for individual-forest management applications.

## 5. Conclusions

Phylogenetic diversity (PD) and phylogenetic community structure (SES.MPD) showed clear forest-type contrasts in Mt. Gariwang, a representative temperate mountain forest in South Korea, but they reflected different underlying controls. PD was most strongly and negatively associated with community-weighted mean wood density, and a substantial fraction of PD variation was attributable to forest-type-level differences, indicating trait-mediated filtering on retained evolutionary history. In contrast, SES.MPD was primarily regulated by community-weighted mean SLA and species richness, with soil fertility influencing SES.MPD indirectly through SLA, consistent with a soil–trait pathway shaping assembly-related coexistence structure. Joint evaluation of PD and SES.MPD therefore provides a practical basis for forest-scale decision making by distinguishing levers for conserving evolutionary history (maintaining phylogenetically broad composition and avoiding dominance by a narrow trait–lineage subset) versus conserving coexistence structure (monitoring SLA-linked strategies and soil conditions that shift assembly signals).

## Data Availability

The original contributions presented in this study are included in the Supplementary Materials, further inquiries can be directed to the corresponding author, Chang-Bae Lee.

## Supplementary Materials

The following supporting information can be downloaded at: https://www.mdpi.com/article/10.3390/life16020301/s1, Figure S1. Phylogenetic tree used for the analyses in this study; Figure S2. Correlation matrix of the biotic and abiotic variables used in this study. An “×” indicates that the correlation between the two variables is not significant. Abbreviations: MAT, mean annual temperature; MAP, mean annual precipitation; TN, total nitrogen; P, available phosphorus; CEC, cation exchange capacity; SR, species richness; CWM, community weighted mean; SLA, specific leaf area; MH, maximum height; WD, wood density; Figure S3. Conceptual model illustrating the hypothesized effects of explanatory variables on phylogenetic diversity and phylogenetic community structure on Mt. Gariwang, South Korea. Species richness was excluded from the analyses of phylogenetic diversity but included in the analyses of phylogenetic community structure. Abbreviations: SLA, specific leaf area; MH, maximum height; WD, wood density; Table S1. Dataset used for analyses in this study. Abbreviations: MAT, mean annual temperature; MAP, mean annual precipitation; PC1_soil, the first principal component axis derived from a PCA of soil properties; Stand age, stand age class; SR, species richness; CWM, community weighted mean; SLA, specific leaf area; MH, maximum height; Faith’s PD, phylogenetic diversity; SES.MPD, standardized effect size of mean pairwise phylogenetic distance; SA, subalpine stands; QM, *Quercus mongolica* stands; OQ, other *Quercus* spp. stands; OB, other broadleaved stands; PD, *Pinus densiflora* stands; LK, *Larix kaempferi* stands; PK, *Pinus koraiensis* stands; Table S2. Results of a principal component analysis (PCA) of three soil properties associated with soil fertility.
