# Supplementary material for: Decoupled Drivers of Phylogenetic Diversity and Community Assembly Signals Across Forest Types in a Temperate Forest, South Korea
Source: Life (Basel). 2026 Feb 10;16(2):301. doi: 10.3390/life16020301 (PMC12942560; doi:10.3390/life16020301)
Supplement: Supplementary file 1 [file life-16-00301-s001.zip › Supplementary material 1_Lee_02_10_2026.pdf]

Supplementary Material

Decoupled Drivers of Phylogenetic Diversity and Community Assembly Signals across Forest Types in a temperate forest, South Korea

Chang-Bae Lee[1,2,3*]

[1]Department of Forest Resources, Kookmin University, 77 Jeongneungro, Seongbukgu, Seoul 02707, Republic of Korea

[2]Department of Climate Technology Convergence, 77 Jeongneungro, Seongbukgu, Seoul 02707, Republic of Korea

[3]Forest Carbon Graduate School, Kookmin University, 77 Jeongneungro, Seongbukgu, Seoul 02707, Republic of Korea

* Correspondence: kecolee@kookmin.ac.kr (C.-B.L.)

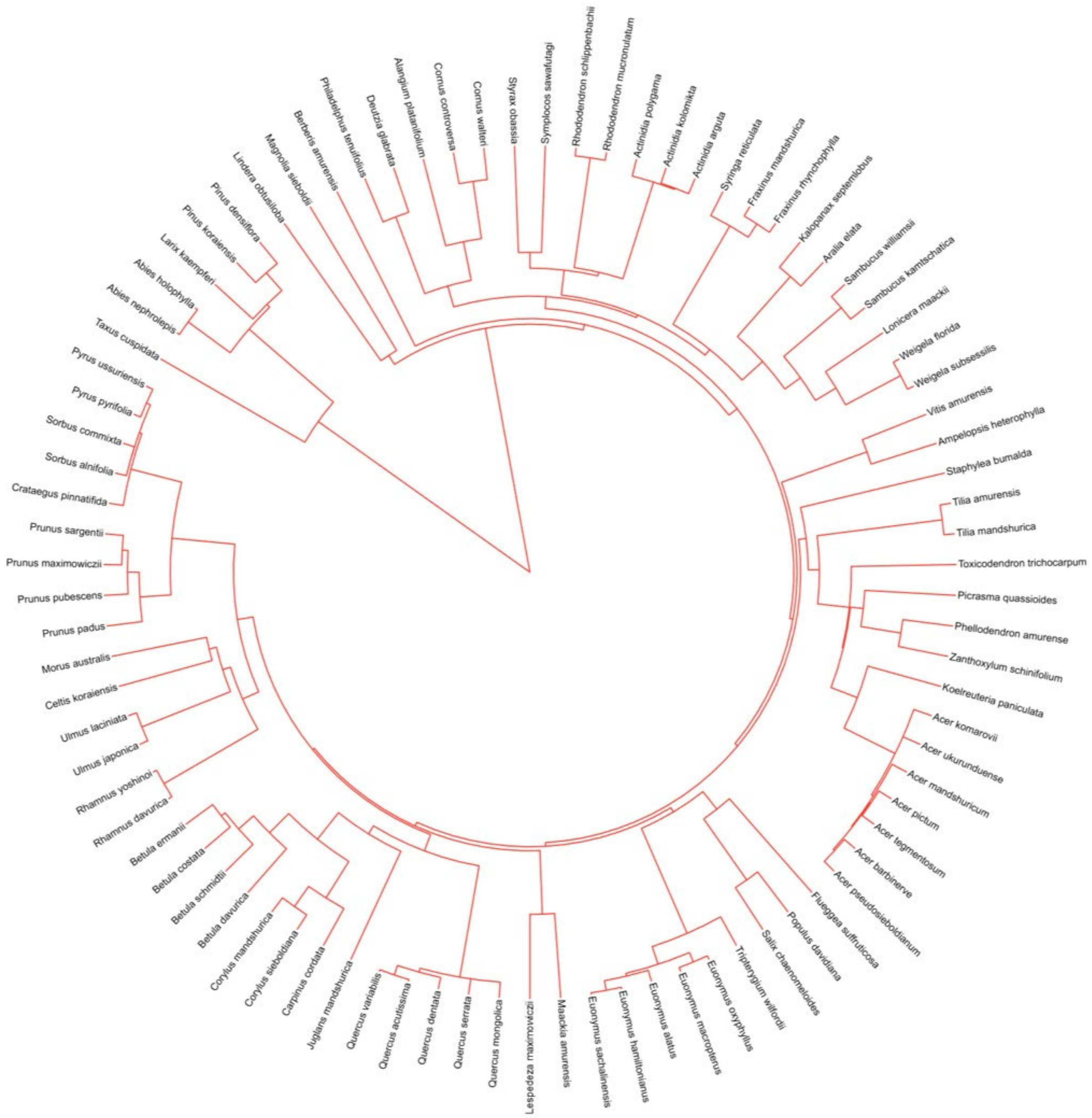

Figure S1. Phylogenetic tree used for the analyses in this study.

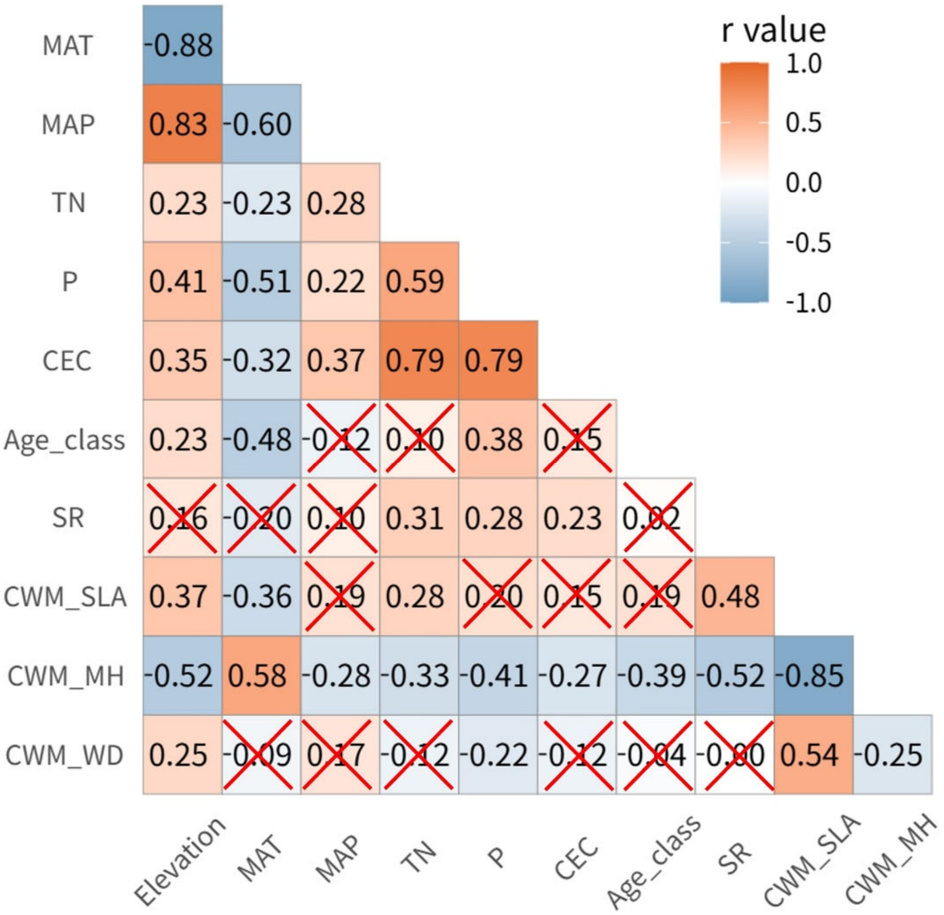

Figure S2. Correlation matrix of the biotic and abiotic variables used in this study. An "×" indicates that the correlation between the two variables is not significant. Abbreviations: MAT, mean annual temperature; MAP, mean annual precipitation; TN, total nitrogen; P, available phosphorus; CEC, cation exchange capacity; SR, species richness, CWM, community weighted mean; SLA, specific leaf area; MH, maximum height; WD, wood density.

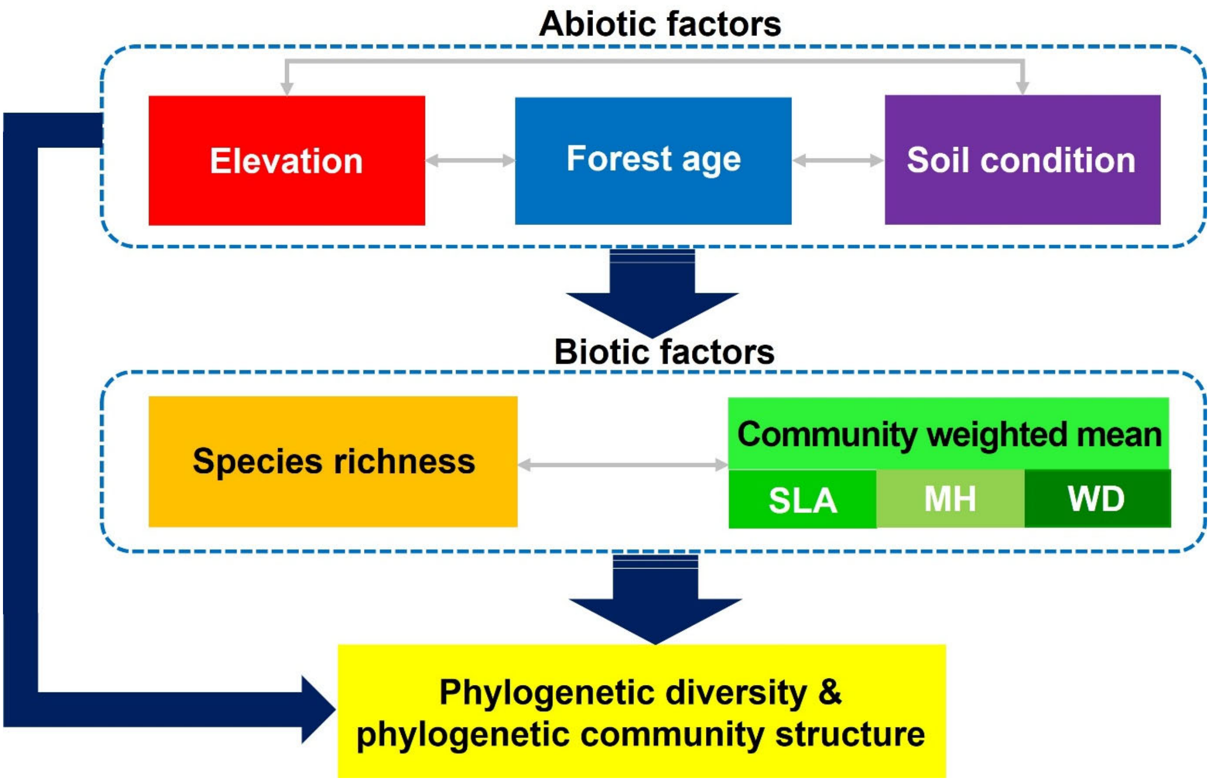

Figure S3. Conceptual model illustrating the hypothesized effects of explanatory variables on phylogenetic diversity and phylogenetic community structure on Mt. Gariwang, South Korea. Species richness was excluded from the analyses of phylogenetic diversity but included in the analyses of phylogenetic community structure. Abbreviations: SLA, specific leaf area; MH, maximum height; WD, wood density.

Table S2. Results of a principal component analysis (PCA) of three soil properties associated with soil fertility.

	PC 1	PC 2	PC 3
Total nitrogen	0.56	–0.71	0.43
Phosphorus	0.56	0.71	0.43
Cation exchange capacity	0.61	0.00	–0.79
Eigenvalue	2.45	0.41	0.14
Variance (%)	81.62	13.71	4.67

R code

1) PCA

```
library(vegan)
library(ggplot2)
library(dplyr)
library(ggrepel)
library(grid)
# Data ------------------------------------------------------------------
df <- read.csv(choose.files(), header = TRUE, fileEncoding = "UTF-8")
# Choose whether to include sesmpd in PCA --------------------------------
include_sesmpd <- TRUE
vars_base <- c("elevation","pc_soil","age","sr","cwm_sla","cwm_wd")
vars <- if (include_sesmpd) c(vars_base, "sesmpd") else vars_base
stopifnot(all(c("FT", vars) %in% names(df)))
# PCA -------------------------------------------------------------------
pca <- rda(df[, vars])
# Scores ----------------------------------------------------------------
site <- as.data.frame(scores(pca, display = "sites", scaling = 2))
load <- as.data.frame(scores(pca, display = "species", scaling = 2))
if (ncol(site) < 2 || ncol(load) < 2) {
  stop("Fewer than two PCA axes were returned. Consider removing highly collinear variables.")
}
colnames(site)[1:2] <- c("PC1","PC2")
colnames(load)[1:2] <- c("PC1","PC2")
load$var <- rownames(load)
site$FT    <- factor(df$FT)
# Optional: prettier variable labels -------------------------------------
name_map <- c(
  elevation = "Elevation",
  pc_soil      = "PC1_soil",
  age             = "Age class",
  sr              = "Species richness",
  cwm_sla      = "CWM.SLA",
  cwm_wd       = "CWM.WD",
```

```
  sesmpd       = "SES.MPD"
)
load$label <- dplyr::recode(load$var, !!!name_map, .default = load$var)
# Rescale arrows -------------------------------------------------------
arrow_mult <- 0.9 * max(abs(c(site$PC1, site$PC2)))
load <- load %>% mutate(PC1 = PC1 * arrow_mult, PC2 = PC2 * arrow_mult)
# Axis labels with explained variance ------------------------------------
eig    <- eigenvals(pca)
labx <- paste0("PC1 (", round(100 * eig[1]/sum(eig), 2), "%)")
laby <- paste0("PC2 (", round(100 * eig[2]/sum(eig), 2), "%)")
# Color palette --------------------------------------------------------
pal <- c(
  "LK" = "#C9B6FF",
  "PD" = "#D8A24A",
  "PK" = "#FDB863",
  "QM" = "forestgreen",
  "OQ" = "#32CD32",
  "OB" = "#00A676",
  "SA" = "#F7A8D9"
)
# Plot ------------------------------------------------------------------
p_pca <- ggplot(site, aes(PC1, PC2)) +
  stat_ellipse(aes(color = FT), type = "norm", linewidth = 0.9, alpha = 0.6) +
  geom_point(aes(color = FT), size = 2.8, alpha = 0.95) +
  geom_segment(
    data = load,
    aes(x = 0, y = 0, xend = PC1, yend = PC2),
    arrow = arrow(length = unit(0.25, "cm")),
    linewidth = 0.9,
    color = "black"
  ) +
  geom_text_repel(
    data = load,
    aes(x = PC1, y = PC2, label = label),
    size = 4.2,
```

```
      color = "black",
      min.segment.length = 0,
      box.padding = 0.3,
      inherit.aes = FALSE
   ) +
   scale_color_manual(values = pal, name = "Forest Type (FT)") +
   labs(x = labx, y = laby, title = "PCA biplot of explanatory variables") +
   theme_minimal(base_size = 13) +
   theme(
      panel.grid.minor = element_blank(),
      axis.title = element_text(face = "bold"),
      plot.title = element_text(face = "bold", hjust = 0.5),
      legend.position = "right",
      legend.title = element_text(face = "bold")
   )
print(p_pca)
# Save figure -----------------------------------------------------------
ggsave("PCA_biplot_FT_colored.jpg", p_pca, width = 8, height = 6, units = "in", dpi = 300, quality = 100)
```

2) PERMANOVA

```
library(dplyr)
library(vegan)
set.seed(123)
# 1) Read data -----------------------------------------------------------
df <- read.csv(choose.files(), header = TRUE, fileEncoding = "UTF-8")
# 2) Select variables ----------------------------------------------------
vars <- c("age","elevation","pc_soil","cwm_sla","cwm_wd","sr")
df2 <- df %>%
   dplyr::select(FT, dplyr::all_of(vars)) %>%
   na.omit()
df2$FT <- as.factor(df2$FT)
# 3) Standardize predictors (recommended) --------------------------------
X <- scale(df2[, vars])
# 4) Distance matrix and PERMANOVA ---------------------------------------
D <- dist(X, method = "euclidean")
```

```
perm <- adonis2(D ~ FT, data = df2, permutations = 9999)
print(perm)
# 5) Check homogeneity of multivariate dispersion ------------------------
bd <- betadisper(D, df2$FT)
print(anova(bd))
print(permutest(bd, permutations = 9999))
# Optional: visualize dispersion
plot(bd)
# 6) Pairwise PERMANOVA ---------------------------------------------------
pairs <- combn(levels(df2$FT), 2, simplify = FALSE)
pair_tab <- do.call(rbind, lapply(pairs, function(pp){
  g1 <- pp[1]; g2 <- pp[2]
  idx <- df2$FT %in% c(g1, g2)
  D_sub <- as.dist(as.matrix(D)[idx, idx])
  fit <- adonis2(D_sub ~ FT, data = df2[idx, ], permutations = 9999)
  data.frame(
    group1   = g1,
    group2   = g2,
    pseudoF = fit$F[1],
    R2        = fit$R2[1],
    p          = fit$`Pr(>F)`[1]
  )
}))
pair_tab$p_adj <- p.adjust(pair_tab$p, method = "holm")
pair_tab <- pair_tab[order(pair_tab$p_adj), ]
print(pair_tab)
# 7) Pairwise dispersion tests (optional) --------------------------------
pair_tab2 <- do.call(rbind, lapply(pairs, function(pp){
  g1 <- pp[1]; g2 <- pp[2]
  idx <- df2$FT %in% c(g1, g2)
  D_sub <- as.dist(as.matrix(D)[idx, idx])
  fit <- adonis2(D_sub ~ FT, data = df2[idx, ], permutations = 9999)
  bd_sub   <- betadisper(D_sub, df2$FT[idx])
  bd_p <- permutest(bd_sub, permutations = 9999)$tab[1, "Pr(>F)"]
  data.frame(
```

```
      group1    = g1,
      group2    = g2,
      pseudoF = fit$F[1],
      R2          = fit$R2[1],
      p            = fit$`Pr(>F)`[1],
      disp_p    = bd_p
  )
  }))
  pair_tab2$p_adj <- p.adjust(pair_tab2$p, method = "holm")
  pair_tab2 <- pair_tab2[order(pair_tab2$p_adj), ]
  print(pair_tab2)
```

3) Multimodel Inference

```
library(sf)
library(MuMIn)
library(nlme)
library(lme4)
library(dplyr)
# 0) Data ----------------------------------------------------------------
data_total <- read.csv(choose.files(), header = TRUE, fileEncoding = "UTF-8")
# Ensure forest type factor (ft)
if ("FT" %in% names(data_total) && !("ft" %in% names(data_total))) {
  data_total$ft <- factor(data_total$FT)
} else if ("ft" %in% names(data_total)) {
  data_total$ft <- factor(data_total$ft)
} else {
  stop("Forest type column (FT or ft) is missing.")
}
# Required columns and type conversion
req <- c("X","Y","pd","elevation","age","phos","tot_nitro","cec","pc_soil","sr","cwm_sla","cwm_wd")
miss <- setdiff(req, names(data_total))
if (length(miss) > 0) stop("Missing columns: ", paste(miss, collapse = ", "))
num_cols <- c("X","Y","pd","elevation","age","phos","tot_nitro","cec","pc_soil","sr","cwm_sla","cwm_wd")
data_total[num_cols] <- lapply(data_total[num_cols], function(x) suppressWarnings(as.numeric(x)))
# Convert lon/lat to UTM (meters) and rescale to km -----------------------
```

```r
# X = longitude, Y = latitude
sf_ll <- st_as_sf(data_total, coords = c("X","Y"), crs = 4326)
bb    <- st_bbox(sf_ll)
ctr <- c((bb["xmin"] + bb["xmax"])/2, (bb["ymin"] + bb["ymax"])/2)    # lon, lat
utm_zone <- floor((ctr[1] + 180)/6) + 1
epsg <- ifelse(ctr[2] >= 0, 32600 + utm_zone, 32700 + utm_zone)
sf_utm <- st_transform(sf_ll, epsg = epsg)
coords <- st_coordinates(sf_utm)
data_total$X_m    <- coords[,1]
data_total$Y_m    <- coords[,2]
data_total$X_km <- data_total$X_m / 1000
data_total$Y_km <- data_total$Y_m / 1000
# 1) Global model (ML for AIC comparison) ---------------------------------
model_lme_total <- lme(
   pd ~ elevation + age + pc_soil + cwm_sla + cwm_wd,
   random          = ~ 1 | ft,
   data              = data_total,
   method          = "ML",
   correlation = corExp(form = ~ X_km + Y_km | ft, nugget = TRUE)
   # , weights = varIdent(form = ~ 1 | ft)
)
# 2) Dredge -------------------------------------------------------------
op <- options(na.action = "na.fail")
dd <- dredge(model_lme_total, trace = FALSE)
# 3) Select models with ΔAIC ≤ 2 ------------------------------------------
best_models <- get.models(dd, subset = delta <= 2)
if (length(best_models) == 0) {
   stop("No models meet the criterion ΔAIC ≤ 2. Consider relaxing the threshold or checking missing data.")
}
# 4) Model averaging -----------------------------------------------------
avg_model <- model.avg(best_models)
# 5) Variable importance and coefficient summary -------------------------
importance_total <- sw(avg_model)
avg_model_total    <- summary(avg_model)
print(importance_total)
```

```
print(avg_model_total)
# Optional: confint(avg_model)
# 6) R² (marginal/conditional) for the global model ----------------------
r2_vals <- MuMIn::r.squaredGLMM(model_lme_total)
print(r2_vals)
```

4) pSEM

```
library(piecewiseSEM)
library(car)
library(dplyr)
library(nlme)
# Data ---------------------------------------------------------------------
data_all <- read.csv(choose.files())
# Select variables and remove rows with missing values ---------------------
vars <- c("sr","cwm_sla","cwm_wd","pd","elevation","pc_soil","age","FT","X","Y")
data_all <- na.omit(data_all[, vars])
# Quick check of correlations among exogenous variables --------------------
round(cor(data_all[, c("elevation","pc_soil","age")]), 3)
# pSEM: full model ---------------------------------------------------------
pSEM_full <- psem(
  lme(cwm_sla ~ elevation + pc_soil + age,
      data = data_all,
      random = ~1 | FT,
      correlation = corExp(form = ~ X + Y, nugget = TRUE)),
  lme(cwm_wd    ~ elevation + pc_soil + age,
      data = data_all,
      random = ~1 | FT,
      correlation = corExp(form = ~ X + Y, nugget = TRUE)),
  lme(pd          ~ elevation + pc_soil + age + cwm_sla + cwm_wd,
      data = data_all,
      random = ~1 | FT,
      correlation = corExp(form = ~ X + Y, nugget = TRUE)),
cwm_sla %~~% cwm_wd,
  elevation %~~% pc_soil,
  elevation %~~% age,
```

```
  pc_soil %~~% age
)
summary(pSEM_full)
dSep(pSEM_full)
# pSEM: reduced model ------------------------------------------------------
pSEM_reduced <- psem(
  lme(cwm_sla ~ pc_soil,
      data = data_all,
      random = ~1 | FT,
      correlation = corExp(form = ~ X + Y, nugget = TRUE)),
  lme(pd         ~ cwm_wd,
      data = data_all,
      random = ~1 | FT,
      correlation = corExp(form = ~ X + Y, nugget = TRUE)),
elevation %~~% pc_soil,
  elevation %~~% age,
  pc_soil %~~% age
)
summary(pSEM_reduced)
dSep(pSEM_reduced)
```
